# The dark side of mobile work during non-work hours: moderated mediation model of presenteeism through conservation of resources lens

**DOI:** 10.3389/fpubh.2024.1186327

**Published:** 2024-02-19

**Authors:** Woo-Sung Choi, Seung-Wan Kang, Suk Bong Choi

**Affiliations:** ^1^College of Business, Gachon University, Seongnam, Republic of Korea; ^2^College of Global Business, Korea University, Sejong, Republic of Korea

**Keywords:** mWork, presenteeism, sleep deprivation, exchange ideology, conservation of resource theory

## Abstract

Owing to the development of Information and Communication Technology (ICT) and the inevitability of telecommuting in the COVID-19 environment, the boundary between working and non-working hours has become blurred. mWork, that is, ICT-based off-hour work, which has increased through the pandemic, affects employees’ work attitudes, such as presenteeism. Hence, we designed a study to investigate the antecedents and mechanisms of employee presenteeism from the perspective of the conservation of resources theory. We supported our hypothesis using a sample of 325 Korean office workers obtained through three rounds of time-delay surveys. The results show that presenteeism is higher among employees with high mWork. In addition, employees’ mWork increases sleep deprivation and presenteeism, and the exchange ideology of employees reinforces the positive effect of sleep deprivation on presenteeism. Additionally, the higher the level of exchange ideology, the stronger the mediating effect of mWork on presenteeism through sleep deprivation. This study verified the conservation of resources theory by identifying the mechanism by which mWork affects an employee’s life, which in turn affects their work, and provides practical implications for managing productivity loss due to presenteeism.

## 1 Introduction

Recently, organizations have been facing fierce global competition and rapid environmental changes, which have had a great impact on the work and life of their members. In particular, the development of Information and Communication Technology (ICT) has enabled the expansion of workspace and time, resulting in both positive and negative effects (1, 2). In other words, ICT makes it possible to overcome the limitations of time and space, facilitate interaction, promote collaboration, and increase productivity (3). However, from the perspective of workers, the development of ICT can lead to an imbalance between work and life, blurring the boundaries between work and daily life and the roles performed by an individual (4–7). Under such circumstances, the impact of mWork on workers’ lives is becoming increasingly important (8).

mWork refers to the ICT-based off-hour work (8). There is a lack of research on the negative aspects of the ICT-based work environment even though the performance of work outside of working hours enabled by ICT can cause job stress and negative work attitudes from the employee’s perspective. In particular, it has been revealed that changes in the working environment due to the use of ICT can cause technostress, which is related to presenteeism and has recently become an issue (9, 10). Moreover, previous studies have suggested that the use of mobile devices induces sleep deprivation (11–13), which is known to be an important cause of presenteeism (14, 15). Also, using mobile devices at night disrupts sleep, leading individuals to start work in a tired state the next morning (16). Nevertheless, the compulsion for continuous connection, both work-related and non-work-related, provides a motive to keep using mobile devices at night (17). In a ubiquitous environment where individuals’ daily lives take place, their leisure and sleep times are decreased (18). This highlights the dark side of mobile work, contrasting with the efficiency gained through the development of information and communication technology.

Presenteeism refers to a state in which an employee goes to work despite having a health problem and works in a state in which attention or concentration is reduced (19, 20). Employees working long hours in physically and mentally uncomfortable conditions can develop low morale or mental health issues due to stress. Furthermore, such conditions can result in a loss of productivity and a depressed organizational atmosphere which can negatively affect organizations (21, 22). Recently, presenteeism is attracting greater attention with the emergence of the concept of “quiet quitting” (23), which refers to a limited commitment to work at the company, reflected in doing only the minimum assigned tasks and no more, and putting personal resources to work at a minimum. According to longitudinal data on US workers compiled by Gallup (24), 2022 is the year with the lowest level of engagement in the past decade. This trend is the strongest among generation Z and younger millennials, who will play an increasingly large role in organizations in the future (25). The degree of immersion in an individual’s organization is influenced by personal characteristics such as exchange ideology (26).

The implications of presenteeism extend beyond organizational productivity; it significantly affects the quality of life of the employees (27). Thus, understanding its precursors and underlying mechanisms is crucial (28). Several studies on presenteeism have paid attention to the motives or antecedents of presenteeism. Though several studies have focused on this issue, Lohaus and Habermann (29) argued that there is a lack of empirical research explaining the process of reaching the state of presenteeism in a theoretical framework and that more research on it is needed. Addressing this gap, our study explores the interplay of sleep deprivation and exchange ideology in the nexus between mWork and presenteeism, through the lens of Conservation of Resources theory. This theory posits that individuals strive to accumulate and safeguard their resources, experiencing stress, job dissatisfaction, and a profound sense of loss when confronted with potential or actual resource depletion (30). Consequently, they endeavor to minimize resource loss and recoup any losses, actively seeking to bolster their resource reserves (30).

The Job Demand-Resource (JD-R) model, an extension of the Conservation of Resources theory, offers insights into various job performance scenarios (31). It suggests that when job demands exceed the available resources, leading to physical and mental strain, employees’ performance suffers (31, 32). Therefore, to enhance employee quality of life and performance, organizations should focus on balancing job demands with available resources, considering work methodologies and employee characteristics. Our study delves into how factors like mWork, sleep deprivation, and exchange ideology contribute to presenteeism, informed by both the Conservation of Resources theory and the JD-R model.

## 2 Theoretical background and hypotheses

### 2.1 mWork and presenteeism

The advancement of ICT fundamentally changes when, where, and how employees work (33). Being connected to work through mobile devices outside of work hours can potentially pose problems for employees (8). When engaging in mWork, individuals invest their personal time, adapt to and handle interruptions, expend energy addressing these disturbances, and manage multiple tasks simultaneously (34).

mWork enables various types of work to be performed without time constraints and anywhere, easily extending work into non-work domains (35). However, such prolonged work activities limit work recovery, leading to long-term tension, sleep issues, and exhaustion (36, 37). In these circumstances, employees lose the opportunity for adequate rest and fatigue recovery, making it difficult to engage in work.

Overwork is a well-known cause of presenteeism (38). Presenteeism refers to a situation where employees are physically at work but unable to fully concentrate (39). mWork reduces free personal time and increases fatigue, depleting the employee’s work resources and hindering their ability to concentrate on work. Hence, mWork leads to an increase in presenteeism. Therefore, we propose the following hypothesis:

*Hypothesis 1 (H1)*: mWork will have a positive (+) relationship with presenteeism. That is, as mWork increases, presenteeism will increase.

### 2.2 The mediating role of sleep deprivation in the relationship between mWork and presenteeism

Long-term work in a physically or mentally uncomfortable state due to organizational factors can lead to stress, reduced morale, and mental health threats, ultimately having a negative impact on productivity and organizational atmosphere. Previous study found that the more one does not get adequate sleep, the more one loses energy and vitality, which causes emotional exhaustion and fatigue (40).

Lack of sleep reduces job satisfaction and simultaneously causes job burnout (41). The use of mobile devices causes sleep deprivation, which causes fatigue and disease, poor health and presenteeism (11, 15). Presenteeism is forcing yourself to go to work despite being in poor health, and individual psychological symptoms such as worker fatigue, exhaustion, depression, and sleep disorders caused by sleep deprivation, and physical symptoms such as gastrointestinal and cardiovascular diseases, negatively affect work performance, causing presenteeism (42).

Recent studies have empirically demonstrated that sleep deprivation increases presenteeism and emotional problems (43, 44). From the background discussed above, sleep deprivation is predicted to mediate the relationship between mWork and presenteeism. Therefore, we propose the following hypothesis:

*Hypothesis 2 (H2)*: Lack of sleep will positively mediate the relationship between mWork and presenteeism. In other words, mWork will increase employee presenteeism by increasing sleep deprivation.

### 2.3 Moderating role of exchange ideology

Exchange ideology refers to the degree of individual belief that work efforts should differ depending on the degree of treatment received from an organization (45, 46). Employees with low exchange ideologies are less sensitive to the organization’s treatment, and the degree of effort they put into work does not change significantly. However, employees with high exchange ideologies do not work hard if they feel that the organization’s treatment is bad or unfair. Johns (20) revealed that the relationship between health status and presenteeism is regulated by variables such as organizational fairness perceived by employees and attitude toward work.

In particular, exchange ideology is becoming an important factor in understanding GenZ and millennial young employees, who are occupying an increasing weight in the organization and establishing the management direction of the organization (25). As the term “quiet quitting” implies, the phenomenon of limited participation and immersion in work at a company, doing only the minimum assigned work and no more, and trying to put the individual’s resources to the minimum is rampant in workplaces. It is influenced by personal characteristics such as exchange ideology (47).

Previous research indicates that sleep deprivation in employees depletes self-regulatory resources, increasing deviant and unethical behaviors at work. However, this process is influenced by individual control motives and efforts, such as self-control, perceived power, goal orientation, and social influence. Subjective norms, a key component of social influence, are formed by the social pressure of reference groups and the motive to conform to their intentions. High exchange ideology indicates a tendency to respond based on one’s subjective reward perception rather than conforming to the group’s intentions. Therefore, from the perspective of Conservation of Resources theory, the relationship between sleep deprivation and negative organizational behaviors like the presenteeism is influenced by the degree of an individual’s transactional attitude in deciding whether to expend resources on self-regulation.

Based on this theoretical background and previous research, it can be hypothesized that employees with a high exchange ideology are likely to perceive the increase in sleep deprivation due to mWork as less fair and will expend fewer resources for self-regulation against negative behaviors resulting from sleep deprivation. Thus, as exchange ideology increases, the relationship between sleep deprivation and an increase in presenteeism is likely to be strengthened. Therefore, we propose the following hypothesis:

*Hypothesis 3 (H3)*: The exchange ideology will statically regulate the relationship between sleep deprivation and presenteeism. In other words, the higher the exchange ideology level, the stronger the effect of sleep deprivation on presenteeism.

### 2.4 Moderated mediation model of exchange ideology

Summarizing the above hypotheses, it can be said that mWork increases sleep deprivation, and increased sleep deprivation increases presenteeism. In this process, exchange ideologies play a controlling role. Exchange ideology is an employee’s sensitivity to an organization’s treatment (26), and employees with high exchange ideology respond with less effort and commitment if they feel that they are being treated unfairly by the organization (47).

Sleep deprivation due to mWork is an instance of poor treatment received from the organization by employees, which depletes individual job resources. Therefore, exchange ideology reinforces the effect of mWork in increasing presenteeism through sleep deprivation. As the level of exchange ideology increases, sleep deprivation, which acts as a parameter in the relationship between mWork and presenteeism, also increases, and the static relationship between mWork and presenteeism becomes stronger. Therefore, we propose the following hypothesis:

*Hypothesis 4 (H4)*: The mediating effect of sleep deprivation on mWork and presenteeism depends on the level of exchange ideology, which will positively (+) regulate the mediating effect of sleep deprivation. In other words, the higher the level of exchange ideology, the higher the mediating effect of mWork on presenteeism through sleep deprivation.

**Figure 1:**
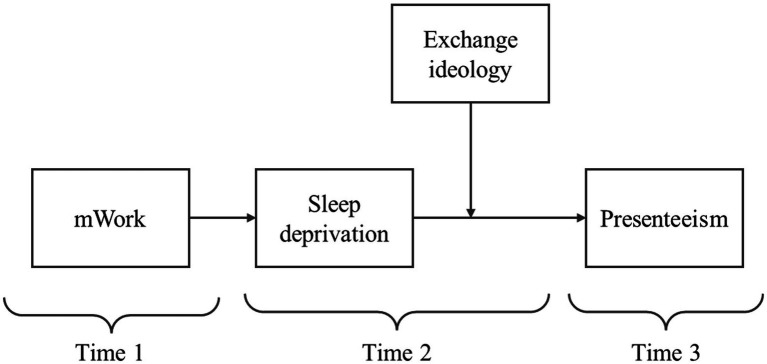
Theoretical model of study. Time 1: mWork; Time 2 (4 weeks after Time 1): sleep deprivation, exchange ideology; Time 3 (4 weeks after Time 2): presenteeism.

The hypothetical research model is in Figure 1.

## 3 Materials and methods

### 3.1 Sample

In this study, to minimize the bias of the common method that may occur due to the cross-sectional survey (48, 49), the survey was conducted in three rounds by dividing the variables with a time difference of one month (50). The survey subjects were randomly selected from an online panel composed of office workers working with bosses in Korean companies. Before responding to the survey, the purpose and procedure of the study, the freedom to withdraw from the survey at any time, and the benefits and disadvantages that may occur when participating were explained to the participants. Thereafter, we asked them to sign an informed consent form and collected data only from respondents who signed it.

The first survey was conducted with 1,200 people via e-mail in December of 2022, and a total of 672 responses were obtained, excluding unreliable responses. In January of 2023, the second survey was sent via e-mail to 672 respondents who had completed the first survey and a total of 450 responses were obtained, excluding unreliable responses. In February of 2023, the third questionnaire was sent to the 450 respondents who had completed the second questionnaire and a total of 325 responses were obtained, excluding unreliable responses. We examined whether there were statistically significant differences in gender and tenure among participants in Waves 1, 2, and 3. Our analysis revealed no significant differences in gender and tenure across the participant groups in Wave 1, 2, and 3. Therefore, we can infer that the likelihood of sample bias introduced by dropouts was minimal.

Of the 325 respondents analyzed in this submission, 50.2% were female and 49.8% were male. The mean age of the respondents was 41.8 years (SD = 11) years. The final education level was four years for university graduates (53.2%), two years for college graduates (20.9%), high school graduates (18.8%), master’s graduates (5.6%), and PhD holders (1.5%). The average tenure at the current company was 7.7 years (SD = 6.8). 55% of respondents were married and 45% were unmarried.

### 3.2 Measures

The participants graded the survey items for the research variables using a five-point Likert scale (1 = strongly disagree, 5 = strongly agree). The measurement items were originally written in English, and after being translated into Korean, they were subjected to professional review and correction. The validity of the Korean survey was, thereafter, verified through reverse translation into English, where the resemblance of linguistic structure and meaning with the original text was contrasted (see Appendix) (51).

#### 3.2.1 mWork

We used the three items developed by Ferguson et al. (8) to assess the frequency with which individuals engage in mWork during off-hours. An example of a question item is, “I often go to work after work or on weekends using a smartphone or a laptop computer.” Cronbach’s alpha was 0.92.

#### 3.2.2 Sleep deprivation

We used the four items developed by Barnes et al. (52) to assess sleep deprivation, indicating poor sleep quantity and quality. An example of a survey item is, “I wake up feeling tired and exhausted after sleeping as much as usual.” Cronbach’s alpha was 0.83.

#### 3.2.3 Exchange ideology

We used the four items developed by Redman and Snape (46) to assess exchange ideology. An example of a question item is, “The degree of effort of members should depend on the degree to which the organization treats them.” Cronbach’s alpha was 0.87.

#### 3.2.4 Presenteeism

We used two items developed by Johns (53) to assess presenteeism. An example item is “Looking back on the past 6 months, did you often go to work without being able to rest at home even if you were not feeling well?” Cronbach’s alpha was 0.92.

#### 3.2.5 Control variable

To confirm the relationship between the variables presented in the research model, female, age, educational background, tenure of employment, and marital status were used as control variables by referring to previous studies dealing with similar research variables (54).

### 3.3 Common method bias

To minimize the possibility of common method bias, the survey was conducted over three rounds with a time difference, and all responses were measured from all rounds of valid respondents. As a result of performing Harman’s single factor test on the survey result (*n* = 325), which is the subject of analysis of this manuscript, the ratio of the first factor was 30.50%, indicating that the research data did not suffer from the serious problem of common method variance (55).

### 3.4 Analysis strategy

We performed CFA to determine model validity and hierarchical regression analysis to test the study hypotheses using STATA 17.0 (Stata Corp., College Station, TX, United States). We followed Hayes’s (56) recommendations when using the bootstrapping approach to test the mediating and moderated mediation hypotheses.

## 4 Result

### 4.1 Descriptive statistics

The means, standard deviations, correlations, and Cronbach’s alpha values are presented in Table 1. It can be confirmed that there are significant correlations between the research variables, consistent with our hypothesis.

**Table 1:** Means, standard deviations, and correlations.

Variables	Mean	SD	1	2	3	4	5	6	7	8	9
1. Female	0.50	0.50	–								
2. Age	41.78	10.97	−0.09	–							
3. Education	2.50	0.92	−0.14*	0.03	–						
4. Tenure	7.67	6.84	−0.12*	0.44***	0.01	–					
5. Marital status	0.55	0.50	−0.13*	0.47***	0.10	0.27***	–				
6. mWork	2.45	1.04	−0.05	0.06	0.21***	0.07	0.08	(0.92)			
7. Sleep deprivation	2.94	0.88	0.12*	−0.07	−0.12*	−0.07	−0.04	0.15**	(0.83)		
8. Exchange ideology	3.52	0.78	0.13*	−0.23***	0.09	−0.19**	−0.13	−0.05	0.09	(0.87)	
9. Presenteeism	2.76	1.10	0.18**	−0.04	−0.10	0.09	−0.03	0.29***	0.35***	0.15**	(0.92)

*n* = 325; *p < 0.05, **p < 0.01, ***p < 0.001 (two-tailed); the values in parentheses denote Cronbach’s alphas; Female, male = 0, female = 1; Age = years; Education = highest education level achieved: 1 = high school graduates, 2 = 2 years of college graduates, 3 = 4 years of university graduates, 4 = master’s graduates, 5 = Ph.D. holders; Tenure = organizational tenure (year); Marital status, unmarried = 0, married = 1.

### 4.2 Confirmatory factor analysis

Table 2 shows that confirmatory factor analysis was conducted to verify the construct validity of the study variables. The resultant chi-square/degree of freedom was 1.74, which is less than the cutoff value of 3.00, and the comparative fit index (CFI) and Tucker Lewis index (TLI) were 0.98 and 0.97, respectively (0.95 is the preference criterion) (57). In addition, the Root Mean Square Error of Approximation (RMSEA) was 0.04, which is lesser than the minimum standard of 0.08 and lower than the preferred standard of 0.05 (57). Judging by the goodness-of-fit index, the goodness of fit of the 4-factor model assumed in this study was very good. By setting and comparing three alternative models, it was confirmed that the 4-factor model was the most appropriate. AVE (Average Variance Extract) and CR (Composite Reliability) values of all variables satisfied the standard values (AVE > 0.5, CR > 0.7), and the correlation coefficient between each variable was lower than the square root of AVE (58). Additionally, all standardized factor loadings of the predictors had a cutoff of 0.50 or higher (58).

**Table 2:** Model fit statistics for the measurement models.

Model	χ^2^(df)	CFI	TLI	RMSEA	Δχ^2^(Δdf)
Hypothesized four-factor model	143.54(88)***	0.976	0.966	0.044	
Alternative 1 (three-factor model)^a^	523.52(96)***	0.816	0.759	0.117	379.98(8)***
Alternative 2 (two-factor model)^b^	1,026.83(103)***	0.603	0.514	0.166	883.29(15)***
Alternative 3 (one-factor model)^c^	1,764.67(109)***	0.288	0.177	0.217	1,621.13(21)***

*n* = 325, ***p < 0.001.

a Three-factor model with sleep deprivation and presenteeism on the same factor.

b Two-factor model with sleep deprivation, exchange ideology and presenteeism on the same factor.

c One-factor model with mWork, sleep deprivation, exchange ideology and presenteeism on the same factor.

CFI, comparative fit index; TLI, Tucker-Lewis index; RMSEA, root mean square error of approximation.

### 4.3 Hypothesis testing

Hierarchical multiple regression analysis was performed to verify Hypotheses 1 and 3, and Hypotheses 2 and 4 were verified using bootstrapping (56). First, as shown in Model 4 in Table 3, there was a significantly positive (+) relationship between mWork and presenteeism (β = 0.33, *p* < 0.001), and Model 4 was significantly more explanatory than Model 3 (Model 3, ➔ Model 4: ΔR^2^ = 0.11, ΔF = 38.50, *p* < 0.001). Therefore, Hypothesis 1 is supported.

**Table 3:** Hierarchical multiple regression results for sleep deprivation and presenteeism.

Variable	Sleep deprivation		Presenteeism			
	Model 1	Model 2	Model 3	Model 4	Model 5	Model 6
Female	0.10	0.10	0.18**	0.18***	0.153**	0.141**
Age	−0.05	−0.06	−0.09	−0.10	−0.08	−0.05
Education	−0.11*	−0.15**	−0.07	−0.14*	−0.09	−0.10
Tenure	−0.04	−0.05	0.15*	0.13*	0.15**	0.14**
Marital status	0.02	0.01	0.01	−0.01	−0.01	0.00
mWork		0.19***		0.33***	0.27***	0.26***
Sleep deprivation					0.29***	0.28***
Exchange ideology						−0.21
Sleep deprivation × exchange ideology						0.12*
R^2^	0.03	0.07	0.05	0.15	0.23	0.26
ΔR^2^		0.04		0.10	0.08	0.02
adj R^2^	0.01	0.09	0.04	0.14	0.22	0.24
F	2.14	3.86***	3.71**	9.87***	13.78***	12.58***
F_inc_		12.10***		38.50***	31.57***	6.65**

*n* = 325, *p < 0.05; **p < 0.01; ***p < 0.001 (two-tailed test). The results are standardized regression coefficients.

Next, 10,000 bootstraps were performed to verify Hypothesis 2. As a result of the bootstrap analysis (see Table 4), which does not depend on the normal sampling distribution assumption, the indirect effect was 0.06. The upper limit of the 95% confidence interval was 0.10 and the lower limit was 0.02; thus, zero was not included in the confidence interval. Therefore, Hypothesis 2 is supported.

**Table 4:** Result of indirect effect test by bootstrapping.

Mediator	Dependent variable: presenteeism			
	Indirect effect	SE	95% CI	
			LLCI	ULCI
Sleep deprivation	0.06	0.02	0.02	0.10

*n* = 325, Bootstrap sample size = 10,000.

SE, standard error; CI, Confidence Interval; LLCI, lower limit of confidence interval; ULCI, upper limit of confidence interval.

**Figure 2:**
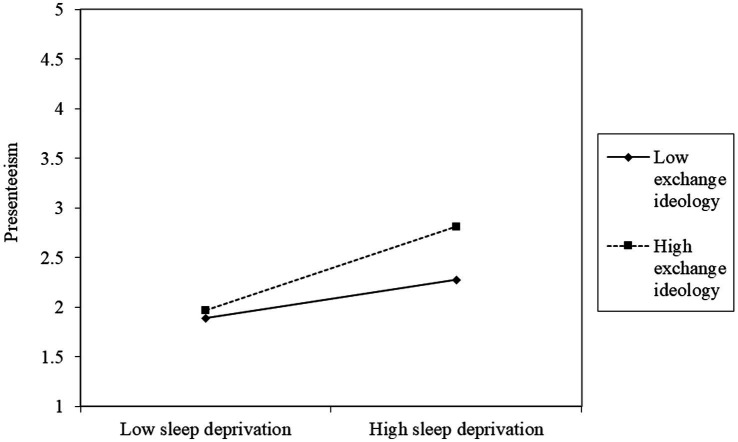
The moderating effect of exchange ideology level on the relationship between sleep deprivation and presenteeism.

Hypothesis 3 is confirmed in Model 6 in Table 3. Presenteeism had a significant relationship with the interaction term of sleep deprivation and exchange ideology (β = 0.12, *p* < 0.05), and the explanatory power of Model 6 was higher than that of Model 5 (Model 5: ➔ Model 6: Δ R^2^ = 0.03, ΔF = 6.65, *p* < 0.01). We illustrated the interaction pattern in Figure 2. Following Aiken and West’s suggestion, we conducted a simple slopes test and the results showed that the positive relationship between sleep deprivation and presenteeism was stronger at a high level of exchange ideology (b = 0.48, *p* < 0.001) than at a low level (b = 0.22, *p* < 0.01) (59). Second, the slopes of the two lines were significantly different (*p* < 0.05) (59). Therefore, Hypothesis 3 is supported.

Finally, Hypothesis 4 was tested. To evaluate the indirect effect, bootstrapping was applied with 10,000 samples, and the indirect effect of mWork on presenteeism through sleep deprivation was estimated at high (+1 SD) and low (−1 SD) levels of exchange ideology. The results in Table 5 show that the indirect effect is stronger at the high level (indirect effect = 0.07, SE = 0.02, 95% CI [0.01, 0.12]) of exchange ideology than at the low level (95% CI [−0.00, 0.06] including zero, not significant). Therefore, Hypothesis 4 is supported.

**Table 5:** Results of conditional indirect effect test by bootstrapping.

Moderator	Level	Dependent variable: presenteeism			
		Indirect effect	SE	95% CI	
				LLCI	ULCI
Exchange ideology	Low (−1 SD)	0.03	0.02	−0.00	0.06
	High (+1 SD)	0.07	0.03	0.01	0.12

*n* = 325, Bootstrap sample size = 10,000.

SD, standard deviation; SE, standard error; CI, confidence Interval; LLCI, lower limit of confidence interval; ULCI, upper limit of confidence interval.

## 5 Discussion

### 5.1 Summary

This study attempted to confirm four hypotheses on the antecedent factors and influence processes of presenteeism from the perspective of the conservation of resources theory. First, we confirmed the relationship between mWork and presenteeism. Moreover, mWork was found to have a positive effect on members’ presenteeism. Second, by examining the mediating role of sleep deprivation in the relationship between mWork and members’ presenteeism, it was found that sleep deprivation mediated the influence of mWork on presenteeism. Third, the moderating effect of exchange ideologies on the relationship between sleep deprivation and presenteeism was verified. Exchange ideologies have been shown to regulate relationships in a positive (+) way. Finally, due to the verification of the moderated mediating effect, it was confirmed that the indirect effect of mWork on presenteeism mediated by sleep deprivation was stronger when the level of exchange ideology was higher.

### 5.2 Theoretical implications

This study contributes to the expansion of resource conservation theory and the identification of the mechanism of presenteeism by presenting several important implications. The conservation of resource theory explains how an individual’s behavior and attitudes toward organizations are influenced by their resource levels in different environments. The COVID-19 pandemic has led to a significant increase in mobile work, including telecommuting and non-face-to-face work. However, if mobile work continues even after the pandemic ends and face-to-face work resumes, it may result in a lack of sleep, which is a crucial personal resource according to the resource conservation theory. Prior research has demonstrated that both work and non-work deviations increase due to constant connectivity to work through information and communication technology, even when not working (60). By applying the theory of resource conservation, this study contributes to the understanding of the relationship between presenteeism and the depletion of personal resources, particularly with regard to mobile work triggered by COVID-19 which continuously affects employee sleep.

First, the relationships between mWork, exchange ideology, sleep deprivation, and presenteeism were verified. Following various previous studies on presenteeism, this study applied the perspective of resource conservation to clarify the structure and relationship of variables based on theory. According to Lohaus and Habermann (29), though many studies tried to find out what the elements related to presenteeism are, most end up listing research results without a theoretical analysis framework or analyzing them based on theory. Moreover, several attempts have been made to theorize inversely by explaining the relationship between variables from the results obtained through analysis. However, it can be seen as a positive phenomenon that research on presenteeism is active and more empirical data are being accumulated (23, 32). This study bridges the existing research gap, expands the theoretical basis for presenteeism, and contributes to the accumulation of in-depth knowledge.

Second, this study revealed the mediating role of sleep deprivation in the relationship between mWork and presenteeism. Engaging in mWork means being simultaneously present in two different spaces and times. That is, one is involved both in the workplace and outside of it, during family time and working hours (61). This blurs the boundaries between work and non-work locations, and between work and personal time, potentially leading to an intrusion into personal life. Indeed, studies on individuals who have experienced remote work during the COVID pandemic have shown that the blending of work and home life in such settings has led to challenges in both domains (62, 63). By revealing that mWork has a great influence on the lives of employees outside of work and can bring about presenteeism through the invasion of personal time and sleep deprivation through the use of mobile devices, an explanation for the other side of work performance using information technology was presented.

Finally, this study contributes to the expansion of Conservation of Resources theory by empirically demonstrating that the relationship between sleep deprivation and presenteeism is moderated by exchange ideology. Individuals strive to acquire and maintain various job resources, but their response to factors that deplete these resources can manifest as presenteeism, with the relationship being strengthened as the level of exchange ideology increases. Specifically, this research proposes that the extent to which an employee’s sleep deprivation manifests as negative behavior toward the organization varies based on the individual’s input of self-regulation resources (64, 65), which can differ according to their level of exchange ideology. Additionally, the study explains within the framework of Conservation of Resources theory how exchange ideology moderates the entire mechanism by which mWork increases presenteeism through sleep deprivation.

This demonstrates that in a management environment where there is an increased emphasis on fairness and heightened sensitivity to resource depletion, exchange ideology, closely related to gender and age, acts as a moderating variable in the relationship between sleep deprivation and presenteeism caused by mWork. This empirical evidence of the role of exchange ideology extends the application of Conservation of Resources theory, providing cues for follow-up studies.

### 5.3 Practical implications

In modern organizations, presenteeism is an important management factor related to the quality of life of employees and the productivity of the organization. According to previous studies, presenteeism leads to a decrease in individual productivity and work ability (66). Also, presenteeism can lead to low job satisfaction and work engagement (67). Regarding the impact of presenteeism on organizations, there is empirical evidence of the hidden costs of lost productivity (68). Our research examines mWork, sleep deprivation, and exchange ideology as antecedent factors of presenteeism and identifies the mechanisms through which each of these variables influences presenteeism. This provides valuable insights for managers and human resource personnel, offering clues to devise practical strategies for reducing presenteeism in the workplace.

The practical implications of this study are as follows. Firstly, by empirically demonstrating that mWork can be a precursor to presenteeism in the context of job resources, this research advises organizations on the efforts needed to reduce presenteeism among their members. High levels of mWork can deplete an employee’s work resources, leading to increased presenteeism, which significantly affects organizational performance. This study particularly highlights the mediating role of sleep deprivation in the relationship between mWork and presenteeism. This suggests that sleep deprivation, as a consequence of mWork, is not only an outcome variable but also a factor that leads to the depletion of work resources. Therefore, mWork management that reduces employees’ commuting time and ensures adequate sleep, thereby improving individual quality of life and preventing organizational productivity loss, is necessary.

Secondly, this research illustrates how exchange ideology modulates the relationship between mWork, sleep deprivation, and presenteeism, taking into account the characteristics of the younger generation, which is increasingly significant within organizations. This implies that the rise in presenteeism could have a more substantial impact on younger generations, necessitating practical solutions. Specifically, it provides important insights into maintaining the benefits of ICT while minimizing its adverse effects. For example, it is crucial to assess whether mWork is essential for each department and task, and apply policy and technical restrictions outside of necessary work hours.

Finally, the increase in internet and mobile device usage due to advancements in information and communication technology has highlighted issues with night work and sleep deprivation. Continuous work and communication with supervisors and clients based on internet and mobile devices lead to increased night work for employees. The rise in night work makes it difficult for workers to get sufficient sleep (69), which can have severe health implications, such as weakened immune systems, increased stress, memory impairments, and decreased concentration (70). Therefore, businesses and governments should regulate working hours and workloads, and limit connectivity outside work hours. For instance, France introduced the ‘Right to Disconnect Law’ in 2016 to ensure rest periods for workers (71). Companies can also help balance employees’ personal lives and work by considering new methods of working and altering work regulations and environments (72). From this perspective, this study presents important managerial implications for businesses and governments.

### 5.4 Limitations and directions for future research

Although this study provides meaningful implications for both scholars and practitioners, several points can be addressed in future research. First, this study has some limitations of sampling method and scope. Although the research data obtained through three surveys with a time difference were used, there are limitations as a cross-sectional study because the measurement of each research variable was limited to individual time points. Therefore, future studies should consider a longitudinal study design. Also, this study analyzed the data measured through an online survey. In the future, other research methods, such as experiments and observations, may be employed to prove a more convincing causal relationship between variables.

Second, this study is a survey of Korean employees, it is possible that their perceptions and attitudes are influenced by their cultural background. Therefore, care must be taken when interpreting these findings and applying them to other countries and cultures. Additionally, future research on the impact of mWork on sleep deprivation and presenteeism in countries with cultural differences could enable comparisons and interpretations from new perspectives or generalizations in different cultural contexts. For example, a recent study targeting workers in New Zealand, which is expected to have cultural differences from Korea, showed that mWork increased work–family conflict among employees, consequently leading to higher turnover intention (73). Work–family conflict can cause stress, which may be linked to sleep deprivation and presenteeism. Additionally, the influence of exchange ideology in South Korea, a culture that emphasizes a sense of duty and hierarchical obedience within organizations, may differ from other countries or cultural backgrounds. The level of self-regulatory resources allocated to mitigate the effects of mWork on personal life intrusion and health issues, leading to presenteeism, can vary depending on the cultural context. Investigating how different cultural backgrounds might influence the modulation effects of various factors, including exchange ideology, on the relationship between mWork and outcome variables, also provides opportunities for diverse follow-up studies.

Third, considering the emphasis of our study on individuals either actively engaged in or having the potential for mobile work (mWork), the procurement of a sample from South Korea is deemed highly pertinent. This pertinence is attributed to the prevalent ownership of personal ICT devices among the working populace in this locale, as demonstrated by a 97% rate of smartphone utilization among adults, which facilitates continuous connectivity for professional purposes. The prevalence of mWork in the Korean workforce is notably significant. However, for subsequent research in diverse environmental or national contexts, employing a more refined methodology in sample selection, focusing particularly on the engagement or prospective engagement in mWork, could enhance the efficacy and relevance of the research findings.

Forth, because the measurements of the research variables covered in this study were made from the same source, they are not free from concerns about the bias of the same method. Though the response time is divided by the time delay, there is a limit in that the source of the response is the same. In this study, as a result of confirmatory factor analysis, it was found that the variables of the research model were classified; however, this issue should be considered in future studies.

Fifth, this study suggests that exchange ideologies are a major moderating variable. However, it can be meaningful to confirm the moderating function of various personal characteristics in the relationship between presenteeism and antecedent variables, along with exchange ideology. Therefore, a more sophisticated research model that can reveal the mechanism of presenteeism can be established if various control variables related to individual characteristics are considered in subsequent studies.

Finally, this study utilized a subjective measurement tool to assess sleep deprivation by relying on participants’ self-reported judgment. This method was deemed appropriate since it closely aligns with individual sleep deprivation experiences, considering that each person may have varying effects on their absolute sleep time. However, it has limitations as it may mistake fatigue caused by factors other than sleep for lack of sleep, and the subjective nature of responses hinders comparison with other individuals. Nonetheless, the subjective measurement was considered the most effective approach for this study, supported by previous research that found a correlation between presenteeism and productivity loss among those subjectively experiencing lack of sleep and those diagnosed with a sleep disorder (74, 75).

Other methods for measuring sleep deprivation include semi-subjective approaches that rely on self-report questionnaires to evaluate if an individual’s sleep time meets a recommended standard, as well as objective approaches that involve using medical or wearable devices to measure sleep quantity and quality (75, 76). In some cases, study designs may intentionally induce sleep deprivation by requiring participants to stay awake while being measured (77). Although these methods have their advantages, the subjective measurement tool was most appropriate for this study among the three methods.

## 6 Conclusion

Through this study, the effect of mWork on presenteeism was analyzed from the perspective of the conservation of resources theory. We confirmed the mediating role of sleep deprivation in the relationship between mWork and presenteeism and demonstrated that exchange ideology functions as an important moderating variable in the overall influence process. In particular, the higher the level of exchange ideology, the greater the indirect effect on presenteeism through mWork-mediated sleep deprivation. Presenteeism can cause losses to organizations and individuals and requires efficient management. Therefore, organizations should strive to ensure that members with high exchange ideology can immerse themselves in their work and contribute to the creation of organizational performance through work-life balance. Despite the limitations of this study, its results provide important implications for corporate organizations that need to manage the presenteeism of members and encourage the participation of the younger generation, in particular.

## Data availability statement

The raw data supporting the conclusions of this article will be made available by the authors, without undue reservation.

## Ethics statement

The studies involving humans were approved by Internal Review Board of Gachon University. The studies were conducted in accordance with the local legislation and institutional requirements. The participants provided their written informed consent to participate in this study.

## Author contributions

W-SC is the principal researcher and prepared the first draft of the article. S-WK supervised the study and refined the draft into a publishable article. SC added valuable theoretical and methodological insights based on his knowledge and expertise regarding the topic of this study. All authors contributed to the article and approved the submitted version.

## Appendix. Measurements

**mWork (α = 0.92)** (8)

1. How frequently do you use a mobile device to perform your job during non-work hours?
2. To what extent do you use mobile device to perform your job during non-work hours?
3. How frequently do you use a mobile device to handle some of your work demands during non-work hours?

**Sleep deprivation (α = 0.83)** (52)

1. I have trouble falling asleep.
2. I have trouble staying asleep (including waking up too early).
3. I woke up several times during the night.
4. I woke up after my usual amount of sleep feeling tired and worn out.

**Exchange ideology (α = 0.87)** (46)

1. A person who is badly treated by a organization should give the organization less support.
2. The effort a person puts into the organization should be related to what the organization does for them.
3. If the organization fails appreciate your contribution, you should do less for the organization.

**Presenteeism (α = 0.92)** (53)

1. Over the past six months I have gone to work despite feeling that I really should have taken sick leave due to my state of health.
2. I have continued to work when it might have been better to take sick leave.
